# Ethyl Chloride as a General Anæsthetic
1Read before the South-Western Branch of the British Medical Association on October 7th, 1903.


**Published:** 1904-03

**Authors:** C. Hamilton Whiteford

**Affiliations:** Senior Honorary Anæsthetist to the South Devon and East Cornwall Hospital; Honorary Anæsthetist to the Plymouth Dental Hospital


### ETHYL CHLORIDE AS A GENERAL ANÆSTHETIC.[1]

BY

C. HAMILTON WHITEFORD, M.R.C.S., L.R.C.P.,

*Senior Honorary Anæsthetist to the South Devon and East Cornwall Hospital; Honorary Anæsthetist to the Plymouth Dental Hospital.*

---

ETHYL CHLORIDE, which until recently had been chiefly employed in England for the production of local anæsthesia by freezing, was given by Heyfelder as a general anæsthetic so long ago as 1848, while dental surgeons have for years noticed occasional cases of general anæsthesia when using ethyl chloride to freeze the gum prior to tooth-extraction.

In 1880 ethyl chloride was investigated by a committee of the British Medical Association and was condemned, probably owing to faulty methods of administration. During the last seven years it has been employed with increasing frequency in different parts of the Continent.

McCardie, in an admirably practical paper which appeared in the *Lancet* of April 4th, 1903, was one of the first to demonstrate that chloride of ethyl, *administered from a closed inhaler*, gave results which placed it in the front rank of general anæsthetics.

In the spring of 1903 I began to employ ethyl chloride, but had only written accounts of its administration to work on, since none of my friends had at that time used it as a general anæsthetic. Since then I have administered it to 150 patients, either alone or as a preliminary to ether, and in three cases as a preliminary to chloroform. Two patients developed asphyxial symptoms, probably due to over-dosage, but caused little anxiety as to ultimate recovery.

At the South Devon Hospital at the present time the house surgeons find ethyl chloride very useful and time-saving in cases requiring rapid and short anæsthesia, such as the setting of fractures and minor operations in the wards, especially at night.

[1] Read before the South-Western Branch of the British Medical Association on October 7th, 1903.

In my private work I find ethyl chloride an admirable substitute for nitrous oxide. Infants and old people take it well, my youngest patient being aged 14 days and my oldest 73 years.

### APPARATUS REQUIRED.

Any form of ether inhaler possessing a bag is sufficient. I have given ethyl chloride from an Ormsby, a Rumboll-Birch, and a Clover, the large-bore inhalers being slightly the better because of the smaller tendency to cyanosis.

It is immaterial into which part of the inhaler the liquid is sprayed. I have had equally good results with the anæsthetic sprayed into the facepiece, the upper part of which must contain a sponge, into the body, or into the bag of the inhaler. I usually, on the ground of convenience, spray the liquid into the bag, which is then attached to the inhaler. The rubber of the bag is not injured by ethyl chloride. The body of the inhaler contains 1 to 1½ oz. of ether, or occasionally chloroform, for the continuance of the anæsthesia.

### DOSAGE.

For a child 3 cubic centimetres } sprayed in one dose.
For an adult 5 to 7 cubic centimetres }

For continuing the anæsthesia, 2 or 3 cubic centimetres every two or three minutes, with frequent breaths of air according to the colour of the patient and the state of the pupil, should be given, similarly in fact to administration of ether.

My longest administration of ethyl chloride alone lasted thirty-five minutes and cost nearly four shillings, which is far too expensive for ordinary work.

Ethyl chloride, as usually put up in graduated glass tubes, has little tendency to decompose; McCardie has successfully employed a stock eighteen months old.

### INDUCTION OF ANÆSTHESIA.

Given from an ether inhaler fitted tightly to the face, anæsthesia is usually complete at the end of one minute. If anæsthesia is not complete in one and a half minutes, the reason is that either air is being admitted, or too little ethyl chloride has been used, most probably the former. Struggling is usually absent, but if present is easily controlled. At the end of one to one and a half minutes the patient is snoring gently, with widely-dilated pupils, loss of corneal reflex, and relaxed muscles. A rash, similar to that of ether, quickly appears, and is most marked on the neck and shoulders. When seen for the first time the sudden insensibility with enormous pupils is very striking.

McCardie has estimated the average duration of ethyl chloride anæsthesia to be seventy-one seconds, which, compared with the average duration of nitrous oxide anæsthesia, given by Hewitt as thirty seconds, gives an advantage in favour of ethyl chloride of forty-one seconds. Return of consciousness is rapid and complete.

Like ether, ethyl chloride, from its stimulating properties, occasionally produces a copious flow of saliva and mucus.

### CONTRA-INDICATIONS.

McCardie considers that the only contra-indication to the use of ethyl chloride is narrowing about the larynx, and, with this one exception, I have given it to all kinds of patients for most of the ordinary major and minor operations without the occurrence of dangerous or serious symptoms either during or after anæsthesia.

### CONTINUATION OF ANÆSTHESIA.

When the anæsthesia has to be prolonged I usually pass from ethyl chloride to ether, which is placed beforehand in the body of the inhaler with the indicator at 0. Breaths of air must be given according to the colour of the patient while the indicator is being rapidly advanced to between 2 and 3. The transition from ethyl chloride to ether is much easier and less liable to be marked by partial return of consciousness than is the transition from nitrous oxide to ether.

If chloroform is necessary from the nature of the operation, I either change from ethyl chloride to ether and from ether to chloroform, or pass from ethyl chloride direct to chloroform, commencing with chloroform in the body of the inhaler, the indicator being at 0, and, as soon as ethyl chloride anæsthesia has been induced, remove the bag and gradually advance the indicator to 1 or 1½, according to the state of the patient.

### EXPENSE COMPARED WITH THAT OF NITROUS OXIDE.

Working with nitrous oxide at six shillings per 100 gallons, and allowing seven gallons for each patient, one nitrous oxide anæsthesia costs 5.13 pence, and with ethyl chloride refills at two shillings and ninepence per fifty cubic centimetres, one ethyl chloride anæsthesia costs 4.71 pence—a difference in favour of ethyl chloride of .42 of a penny, or three shillings and sixpence on each hundred administrations.

### RISKS TO LIFE.

No drug sufficiently powerful to produce unconsciousness can be absolutely free from risk. Luke[1] says, "Seitz alone has published records of 12,500 cases with only one death. In all the fatalities which have occurred under ethyl chloride—and there are only four or five cases on record—the patient has been suffering from some grave cardiac or respiratory disability, and as Dr. Hewitt puts it in the September number of the *British Dental Journal*, we are now entitled to consider that 'ethyl chloride is a comparatively safe anæsthetic,' as safe, all things considered, as ether. I should now put the mortality at something between 1 in 10,000 and 1 in 15,000; but of course, as with all anæsthetics, much depends on the experience of the administrator," and later says, "Employed in its own sphere, namely, for inducing anæsthesia only prior to ether or chloroform, and for brief dental or throat operations, ethyl chloride is undoubtedly very much safer and more convenient than chloroform."

### GENERAL CONCLUSIONS.

Ethyl chloride, given for short operations, produces an anæsthesia which lasts more than twice as long as that of nitrous oxide. In short dental operations, ethyl chloride, by producing muscular relaxation, does away with the preliminary gagging, which to many patients is the most objectionable part of nitrous oxide anæsthesia.

[1] *Brit. M. J.*, 1903, ii. 951.

From its portability and power of rapidly inducing a general anæsthesia which is followed by quick recovery and slight after-effects, ethyl chloride bids fair to come into general use, especially in private and country practice, and, as a preliminary to ether or chloroform, appears likely to largely supersede nitrous oxide.

